# What Atomic Positions Determines Reactivity of a Surface? Long‐Range, Directional Ligand Effects in Metallic Alloys

**DOI:** 10.1002/advs.202003357

**Published:** 2021-02-14

**Authors:** Christian M. Clausen, Thomas A. A. Batchelor, Jack K. Pedersen, Jan Rossmeisl

**Affiliations:** ^1^ Department of Chemistry University of Copenhagen København Ø 2100 Denmark

**Keywords:** adsorption energy predictions, electrocatalysis, high‐entropy alloys, ligand effects, oxygen reduction reaction

## Abstract

Ligand and strain effects can tune the adsorption energy of key reaction intermediates on a catalyst surface to speed up rate‐limiting steps of the reaction. As novel fields like high‐entropy alloys emerge, understanding these effects on the atomic structure level is paramount: What atoms near the binding site determine the reactivity of the alloy surface? By statistical analysis of 2000 density functional theory calculations and subsequent host/guest calculations, it is shown that three atomic positions in the third layer of an fcc(111) metallic structure fourth‐nearest to the adsorption site display significantly increased influence on reactivity over any second or third nearest atomic positions. Subsequently observed in multiple facets and host metals, the effect cannot be explained simply through the d‐band model or a valence configuration model but rather by favorable directions of interaction determined by lattice geometry and the valence difference between host and guest elements. These results advance the general understanding of how the electronic interaction of different elements affect adsorbate–surface interactions and will contribute to design principles for rational catalyst discovery of better, more stable and energy efficient catalysts to be employed in energy conversion, fuel cell technologies, and industrial processes.

## Graphical abstract

A statistical analysis uncovers what atomic positions, near the binding site on the surface of an alloy, exert an electronic effect on the adsorbate–surface bond. It is found that a specific position in the third layer has a significantly larger effect on the binding energy than similar or closer positions due to a favorable direction of interaction.

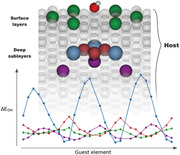

One of the present grand challenges in energy conversion and chemical production is to design catalyst materials in a rational manner. The Sabatier principle states that a good catalyst material is a compromise between binding the reactants while not binding too strongly so that the products will poison the catalyst surface.^[^ ^1^ ^]^ Whereas this provides a nice intuitive way of explaining catalytic activity it does not provide a quantitative goal, which can be used for the design of catalysts. In the descriptor‐based approach to catalyst discovery the whole energetic reaction path is described by a single or few adsorption energies of key intermediates manifested by the scaling relations^[^ ^2^, ^3^ ^]^ and Brøndsted–Evans–Polanyi relations;^[^ ^4^ ^]^ thus obtaining a quantitative goal. This descriptor methodology forms a direct link from adsorption energy to activity and can provide useful design principles for identifying promising catalyst materials.^[^ ^5^, ^6^ ^]^

Different pure metal surfaces might not have the optimal adsorption energy; the right balance between a not too strong and a not too weak binding associated with the highest activity. Therefore, alloys can have a higher activity as the adsorption energies can be perturbed relative to the pure metals by changing the composition of the alloy. The two main effects responsible for this perturbation are the strain effect and the ligand effect. The strain effect is the increase or decrease of adsorption strength by tensile or compressive strain, respectively^[^ ^7^ ^]^ and it has been studied and utilized extensively.^[^ ^8^, ^9^, ^10^ ^]^ The ligand effect arises from the electronic structure of the binding metal atom which is affected by the surrounding metal atoms close to the binding site. The effect of a subsurface layer of a different guest element has been seen to change the activity of the host atoms in the surface.^[^ ^11^ ^]^ This has been explained by different electronic descriptors among those the d‐band^[^ ^12^, ^13^, ^14^, ^15^ ^]^ and the valence configuration.^[^ ^16^ ^]^ One example is the ligand effect on Pt(111) with Cu in the subsurface layer. It has been shown in very well controlled electrochemical experiments that the adsorption energy depends on the amount of Cu in the subsurface and that this has a direct effect on the oxygen reduction reaction (ORR) activity both in acidic^[^ ^17^ ^]^ and alkaline environments.^[^ ^18^ ^]^ The ligand effect is believed to be short‐range^[^ ^19^, ^20^, ^21^ ^]^ and therefore atoms close to the binding site should give a larger effect than atoms further away but beyond this, not much is known about the extent of the ligand effect. Therefore, questions arise: At what atomic positions relative to the binding site does the alloy composition influence the adsorption energy of an intermediate and what other factors are at play apart from range?

Here we show a ligand effect emanating from the fourth nearest neighboring atoms which is observed to be direction‐dependent and long‐range based on density functional theory (DFT) simulations. We find that the effect applies generally among different facets and host/guest elements. For an fcc(111) surface, it is found by statistical analysis of 2000 different high‐entropy alloy (HEA) slabs^[^ ^22^ ^]^ that three specific atomic positions in the third layer below the surface affect the binding on a surface Pt atom more than any second or third nearest positions and in fact as much as the nearest positions in the subsurface. We also show that besides the nearest positions and the aforementioned fourth nearest positions none of the other positions in the atomic structure will give rise to a sizeable ligand effect.

This effect has not previously been described and it is not captured by simple models such as the d‐band model or valence configuration model. The reason this effect has been overlooked up until now is probably that the discovery took particular analysis of many hundred calculations as part of our ongoing research into catalysis on HEAs. However, now identified it is possible to find it for any host/guest system or HEA. Besides revealing a surprising directional effect for bonds to metal surfaces, this discovered effect might also be used to design optimal catalytic surfaces without changing any atoms in the two top layers which are the layers most prone to interact with the environment and therefore most likely to dissolve during reaction conditions. Furthermore, this will enable more accurately predicted binding energy distributions for high‐entropy alloys from which estimates of the catalytic properties can be made.

The framework of this investigation is the intermediates of the oxygen reduction reaction (ORR) on an fcc(111) surface of the equimolar high‐entropy alloy Ir${}_{20}$Pd${}_{20}$Pt${}_{20}$Rh${}_{20}$Ru${}_{20}$ with subscripts indicating percentage‐wise bulk composition. In the following analysis we assume this alloy to be stable and fully miscible as to achieve a completely random placement of atoms of all five elements. Due to the number of local compositions possible in HEAs, the surface is expected to have a characteristic distribution of binding energies which makes these alloys a new possible tool in catalytic applications. Unfortunately, the vast number of local compositions also makes computational simulation of all possible sites practically unobtainable and it is therefore necessary to ascertain the distributions of binding energies from subsets of calculations. It has been shown that the element of the surface atom(s) to which the bond(s) of the intermediate is formed is by far the most dominant feature when determining the binding energy of the adsorbate^[^ ^22^, ^23^ ^]^ as would be expected. We thus limit our analysis to OH adsorbed on‐top on a Pt surface atom and O adsorbed in fcc hollow sites of Ir, Pd, and Pt, as shown in **Figure** **1**, since this will isolate the effects of the local environment on the binding energy of the intermediate (ΔE). We used DFT calculated sets of approximately 1000 ΔEs of OH and O, respectively, on periodical 5 × 5 × 4 atom sized slabs of the equimolar alloy at the lattice parameter corresponding to the average of the five constituent elements. Each layer was kept at an equimolar composition and the elements were randomly assigned within each layer. This represents an unstrained situation where each layer has the bulk composition and therefore also the bulk lattice parameter in accordance with Vegard's law.^[^ ^24^ ^]^ An example of these slabs is shown in Figure S1, Supporting Information.

**Figure 1:**
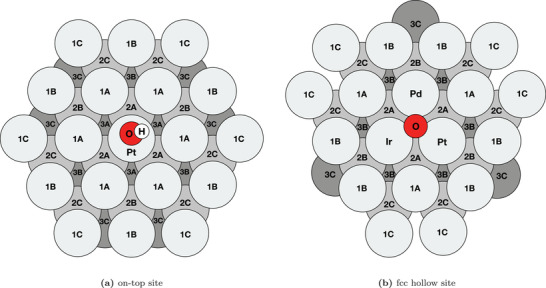
Schematic of atomic positions grouped by layer and distance from the binding site on an fcc(111) surface microstructure for a) on‐top adsorption on Pt and b) fcc hollow site adsorption on IrPdPt. Fourth layer contains zones 4A, 4B, 4C and 4D which has identical layout as the first layer. Note that the two adsorption sites are very different in terms of coordination and distance to the adsorbate and that zones therefore cannot be directly compared between the different types of site.

Ensuring an unstrained environment for the binding site we have now isolated the electronic effect of ligands from the strain effect. Next, we truncate the structure in the DFT dataset by tallying up how many atoms of each element each zone in Figure 1 consists of and denote this in a feature vector as shown in Figure S2, Supporting Information. This provides a simple way of encoding the microstructure of each slab which we deem sufficient to encompass the electronic effects of orbital overlap and links the microstructure features to the DFT calculated adsorption energy. To analyze the perturbation of ΔE of each element in each zone we fit the features to the adsorption energies in a least squares procedure using a multiple linear regression model. The accuracy of the regression model was calculated as the average of a cross validation, yielding a mean average error (MAE) 0.036 eV for on‐top sites and 0.071 eV for hollow sites with further details provided in section, Supporting Information.

The fitted coefficients of the regression model corresponding to each variable in the feature vector are displayed in **Figure** **2** and we observe general trends for both on‐top and hollow site adsorption: As expected, the coefficients of the nearest zones directly coordinated to the binding site have a larger spread than those of the outermost zones, corresponding to larger perturbation of the adsorption energy. Additionally, the effect on ΔE trails of as the distance to the binding site increases. Another factor to consider is that zones containing six atoms are statistically closer to equimolar composition and therefore have less influence on the binding energy echoing the mean field effect.^[^ ^25^ ^]^

**Figure 2:**
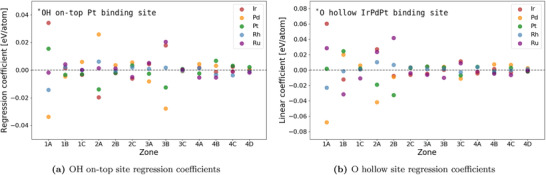
Overview of the regression coefficients of the least squares fits for each element by zone. It can be seen that the atoms with direct coordination (i.e., zones 1A and 2A) to the binding atoms have a large effect on ΔE. Furthermore, zone 3B in the on‐top adsorption scheme has a similar sized impact on ΔE as the subsurface neighbors in zone 2A despite being among the fourth nearest neighboring zones to the binding site. Refer to page S2, Supporting Information, for further details on the fitting procedure.

Curiously, we observe the composition of on‐top zone 3B in the third layer having a large effect while simultaneously observing no sizeable effect from surface or subsurface zones closer to the adsorption site (i.e., zones 1B, 2B, and 2C). This effect was confirmed by ΔE‐calculations of multiple combinations of pure metal host structures with zones comprising different guest elements as seen in **Figure** **3** and Figure S6, Supporting Information. For these calculations, 3 × 4 × 5 atom sized slabs were used as shown in Figure S3, Supporting Information.

**Figure 3:**
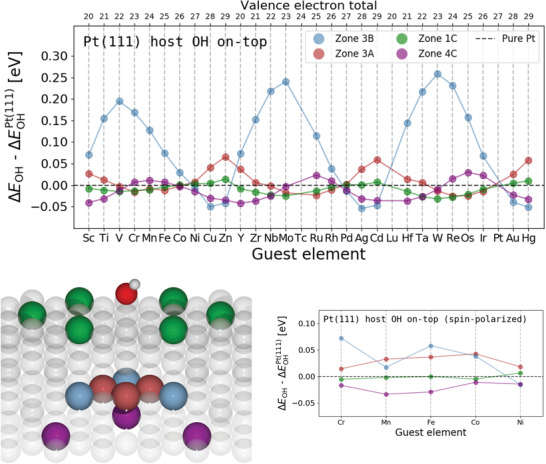
OH adsorption energies on Pt with inserted guest elements in different zones. All adsorption energies are relative to a pure Pt(111) host. The valence electron total is calculated as the sum of valence electrons of one host atom, one guest atom and the adsorbate atoms. All calculations were performed at the same fixed atomic geometry but the results remain similar after relaxation of atomic geometries as shown in Figures S5 and S4, Supporting Information. A schematic drawing with colored atomic positions for each zone are included. Due to slab size restrictions only two‐thirds of the atoms in zone 1C and 4C were exchanged for guest elements whereas all the atoms in zone 3A and 3B where exchanged. Spin‐polarized calculations for the Cr‐Mn‐Fe‐Co‐Ni family of elements are shown in the lower left. The adsorption energies of W in all zones were confirmed with the BEEF‐vdW functional^[^ ^34^ ^]^ as listed in Table S1.

Apparently, this effect also manifests in structures composed of a single host metal and is therefore not a phenomenon related to HEAs in particular. As is evident from Figure 3, there is a clear correlation between the adsorption energy and the number of valence electrons of the guest element. The correlation changes with host element, as shown in Figure S6, Supporting Information, where weakest OH binding are achieved around a total of 17 valence electrons of the host element and guest element combined. This, coupled with the 7 valence electrons of O and H combined, totals to 24 valence electrons which has previously been reported as a particularly stable configuration for near‐surface alloys (NSAs) with an atomic sublayer solely comprised a single guest element by Calle‐Vallejo et al.^[^ ^16^ ^]^ They argue that a total of 24 valence electrons leads to completion of the octet rule for the adsorbate and the 18‐electron rule for the metallic atoms when accounting for the bonds between the first three atomic layers and the adsorbate. This stability in turn leads to a strongly bound adsorbate. Due to the heterogeneous microstructure of our zone model there is no simple way to account for bonds between layers as the lateral and diagonal bonds must be considered as well. Nevertheless, we see the third layer lead to the opposite effect as Calle‐Vallejo observes for the sublayer which points to a possible even/odd‐type effect: A reactive element in the sublayer reduces the reactivity of the surface by binding the top layer tightly to the sublayer and as a consequence the bond to the adsorbate is weakened. Conversely, a reactive element in the third layer binds the sublayer tightly freeing the top layer to bind the adsorbate more strongly. This is also exhibited in the regression coefficients by some guest elements especially Ir and Pd which has completely opposite effects that alternate with each layer.

We observe the effect to scale linearly with the amount of guest atoms in zone 3B as shown in Figure S7, Supporting Information, and be independent of slab size as shown in Figure S8, Supporting Information. Furthermore, although diminished the effect is also present in other facets which also change the interaction depending on the facet as shown in Figure S9, Supporting Information. By examination of the regression coefficients for hollow sites in Figure 2b elements in zone 3C has similar regression coefficients as on‐top zone 3B but of smaller magnitude. A scan of guest elements in hollow site zone 3C reveals that the effect can attain even greater magnitude as evident from Figure S11, Supporting Information. This increased change in reactivity compared to the host/guest calculations of OH are due to the higher order of the O‐surface bond.^[^ ^26^ ^]^ This does not show up in the regression coefficients since the investigated hollow sites are IrPdPt‐sites which will interact with the guests differently and therefore obscure the interaction. The host/guest calculations were generally not spin‐polarized but supplementary calculations including spin‐polarization were carried out for the 3d metals Cr to Ni seen in Figure 3 and Figure S12a, Supporting Information, and all 5d metals as shown in Figure S12b, Supporting Information. We observe that spin‐polarization of calculations partly quenches of the adsorption energy perturbation for the magnetic 3d metals, especially for Cr and Mn, however, the magnitudes of perturbation for the non‐magnetic 5d metals remain unaffected.

Independent of host/guest relationship zone 3A often has a negligible or an opposite effect of zone 3B which can be explained from the added or subtracted electron density from the guest atoms. Figure S13, Supporting Information, displays the electron density of the host slab including the guest atoms where the electron density of the pure host slab has been subtracted. The electron density perturbation can be seen propagating from the guest atoms through neighboring atoms without a significant change of direction. A vector going from any atom in zone 3B to the binding atom passes through an atom in zone 2A and the change in electron density can therefore easily propagate from the third layer to the surface layer as schematized in **Figure** **4**a. On the contrary, no single vector can be drawn through bonds from the atoms in zone 3A to the binding atom and perhaps as a consequence of this these atoms have little effect on ΔE even though their euclidean distances to the binding atom are smaller. The perturbation of the electron density caused by zone 3A will mainly propagate to the atoms in zone 1A instead as schematized in Figure 4b.

**Figure 4:**
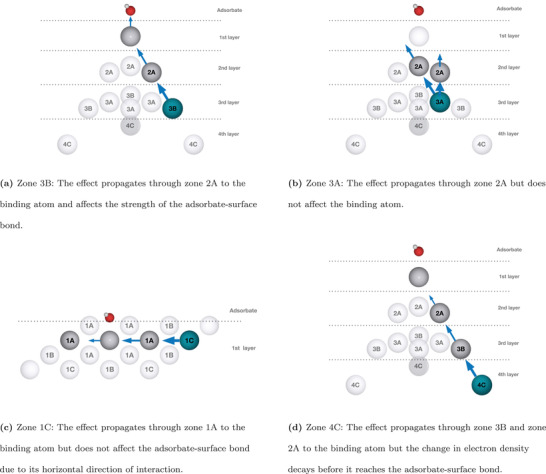
Illustrations of the directional perturbation of electron density from the different atomic positions. These schematic diagrams provide a clear overview of the electron density isosurfaces of which some are displayed in Figure S13, Supporting Information.

It is also possible to draw vectors through bonds from zone 1C and 4C, but as shown in Figure 3 these do not cause any significant effect. Zone 1C causes a similar electron density change on the binding atom as zone 3B. However the perturbation is horizontally directed and therefore perpendicular to the adsorbate‐surface bond as schematized in Figure 4c. Surface and subsurface electronic interactions have previously been deconvoluted from strain and related to d‐band properties^[^ ^27^ ^]^ but to our knowledge, no studies have shown a dependency on whether the electronic effect is laterally or vertically directed. zone 4C is in the fourth layer so the electron density change decays before reaching the binding atom as seen in Figure 4d. This result supports the earlier studies^[^ ^19^, ^20^, ^21^ ^]^ which found the vertical electronic effect fading rapidly with increasing atomic layers.

As a rule of thumb, shifts of the d‐band towards lower energies will cause some of the antibonding orbitals formed between the adsorbate and surface to be pulled below the Fermi level which results in a weaker adsorbate‐surface bond.^[^ ^28^, ^29^ ^]^ From Figure S15a, Supporting Information, we see that the d‐band center is not directly correlated with the changes in adsorption energy as was also found for NSAs^[^ ^16^ ^]^ but by inspection of Figure S15b, Supporting Information, clear trends of small but sequential changes to the d‐band shape are apparent. Changing a whole sublayer can cause notable shifts in the position of the d‐band center and bond strength^[^ ^12^ ^]^ but we see that changing the element of a few atoms in the third layer perturb the shape of the d‐band in a subtler, but still systematic, way which impacts the bond strength on a consequently smaller scale. Even though the change of d‐band shape is not readily quantifiable one could estimate that the increasing projected density around −1.0 eV could increase the overlap with some antibonding orbitals. Conversely, the diminishing density below −1.5 eV could lessen the overlap of bonding orbitals. Both of these sequentially weaken the surface‐adsorbate bond in agreement with the observed bond strength. By comparing the changes of the d‐band caused by guest atoms in zone 3B and G, respectively (Figures S15b and S14b, Supporting Information), we see much less perturbation caused by zone 3A agreeing with the negligible change in adsorption energy.

By inspection of Figure 3, it can be seen that zone 4C has peaks of ΔE shifted towards lower guest valency relative to zone 3B and moreover, that zone 3A and 4C show inverse perturbations hinting at more layers of interaction which will require further investigation. In future works we will investigate more types of adsorbates and facets to fully uncover their influence on the observed long‐ranged effect.

After statistical analysis of 2000 DFT calculations of binding site ensembles occurring on an equimolar HEA we have found that certain atomic positions relative to the binding site of an adsorbate are more prone than others to affect the strength of the bond between adsorbate and surface. This interaction is not a function of the euclidean distance as we observe selected atoms among the fourth nearest neighboring positions having more impact on the bond strength than any second and third nearest positions. This phenomenon is not confined to HEAs or even fcc(111) facets as confirmed with DFT calculations of pure metallic hosts with only a few guest elements in the aforementioned positions. The effect on the binding energy is correlated with the valency difference between the host and guest elements as well as the element and facet of the host. By studying the isosurfaces of electron density differences between these slabs we found that the likely reason for direction dependence of the interaction is the propagation of change in electron density along vectors through bonds in the metallic solid. Finally, the calculations of how the host/guest relationship affects the bonding strength revealed that the changes in bond strength does not correlate with the d‐band center but rather with certain features of the d‐band shape.

These results will enable more accurate ΔE predictions which will improve future theoretical work with HEA surfaces where the bond strengths cannot be computed due to the amount of data needed to fully describe the surface properties. By further investigation of the host/guest/facet‐relations interpolation between these may act as an additional rule of thumb for surface properties and guide our attention towards interesting areas in the large alloy composition space.

## Experimental Section

All density functional theory calculations were performed with the RPBE exchange‐correlation functional^[^ ^30^ ^]^ as this has previously been shown to be adequate for these calculations.^[^ ^22^ ^]^ The calculations were carried out using the atomic simulation environment (ASE)^[^ ^31^, ^32^ ^]^ and GPAW^[^ ^33^ ^]^ using plane‐wave expanded wavefunctions with an energy cut‐off of 400 eV. The slabs were sampled with a Monkhorst‐Pack k‐point sampling of the Brillouin zone of (2 × 2 × 1) and (4 × 4 × 1) for 5 × 5 × 4 atom sized slabs and 3 × 4 × 5 atom sized slabs, respectively. A 7.5 Å vacuum was added above and below the slabs, and atomic layers below second layer were fixed during atomic geometry optimization procedures. Illustrations of slabs and adsorbate locations are shown in Figures S1 and S3, Supporting Information, after relaxation to a maximum force of 0.1 eV Å${}^{-1}$ on all atoms. Adsorption energies were calculated as:

(1) $$\Delta E_{\text{OH}}=E_{\text{slab}+\text{OH}}+\frac{1}{2}E_{H_{2}}-E_{\text{slab}}-E_{H_{2}O}$$

(2) $$\Delta E_{O}=E_{\text{slab}+O}+E_{H_{2}}-E_{\text{slab}}-E_{H_{2}O}$$

with $E_{\text{slab+OH}}$ and $E_{\text{slab+O}}$ being the energy of the relaxed surfaces with adsorbates and $E_{\text{slab}}$ the energy of the relaxed slab without adsorbates. $E_{H_{2}}$ and $E_{H_{2}O}$ are calculated gas‐phase reference energies with identical calculation parameters as the slabs other than the Brillouin zone being solely sampled by the Γ‐point. Calculations of involving Tc and Lu were not included due to unavailable atomic projected augmented wave (PAW) setups.

## Conflict of Interest

The authors declare no conflict of interest.

## Author Contributions

C.C. wrote the main part and carried out the DFT calculations and the modeling. J.R. provided the introduction. C.C., T.B., J.P., and J.R. contributed to the conceptual framework of the methodology.

## Supporting information

Supporting Information

## Data Availability

Data and processing scripts are accessible at https://nano.ku.dk/english/research/theoretical-electrocatalysis/katladb/longrange-directional-ligand-effects-in-metallic-alloys/.
