# Supplementary material for: What Atomic Positions Determines Reactivity of a Surface? Long‐Range, Directional Ligand Effects in Metallic Alloys
Source: Adv Sci (Weinh). 2021 Feb 14;8(9):2003357. doi: 10.1002/advs.202003357 (PMC8097360; doi:10.1002/advs.202003357)
Supplement: Supplementary file 1 — Supporting Information [file ADVS-8-2003357-s001.pdf]

© 2021 Wiley-VCH GmbH

ADVANCED SCIENCE
Open Access

Supporting Information

for *Adv. Sci.,* DOI: 10.1002/advs.202003357

What Atomic Positions Determines Reactivity of a Surface? Long-Range, Directional Ligand Effects in Metallic Alloys

Christian M. Clausen, Thomas A. A. Batchelor, Jack K. Pedersen, and Jan Rossmeisl*

Supplemental Information

What Atomic Positions Determines Reactivity of a Surface? Long-Range, Directional Ligand Effects in Metallic Alloys

Christian M. Clausen, Thomas A.A. Batchelor, Jack K. Pedersen, Jan Rossmeisl

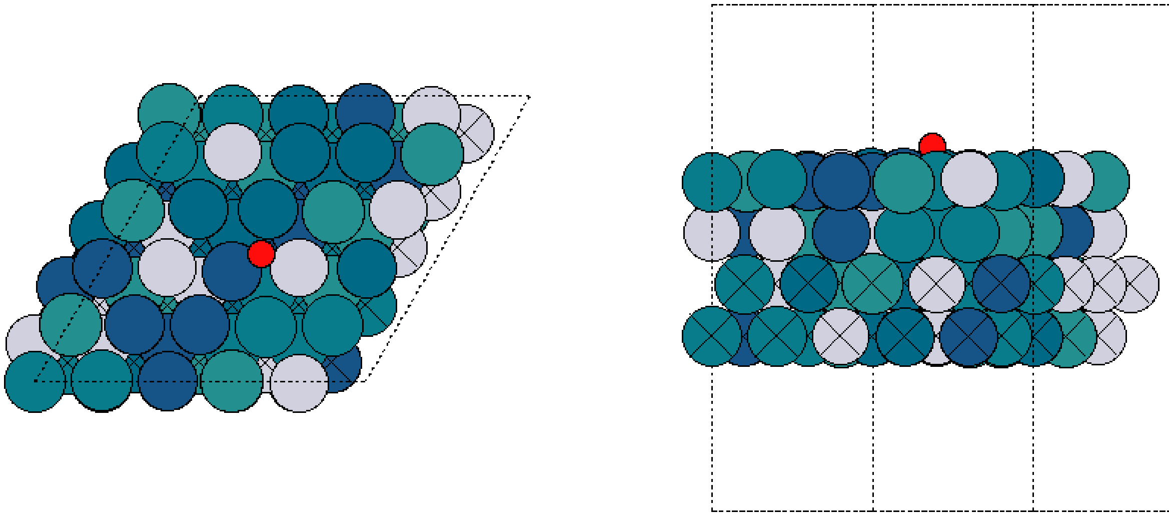

Figure S1: Illustration of adsorbed O(red) in a hollow site on a 5x5x4 slab of the equimolar HEA.

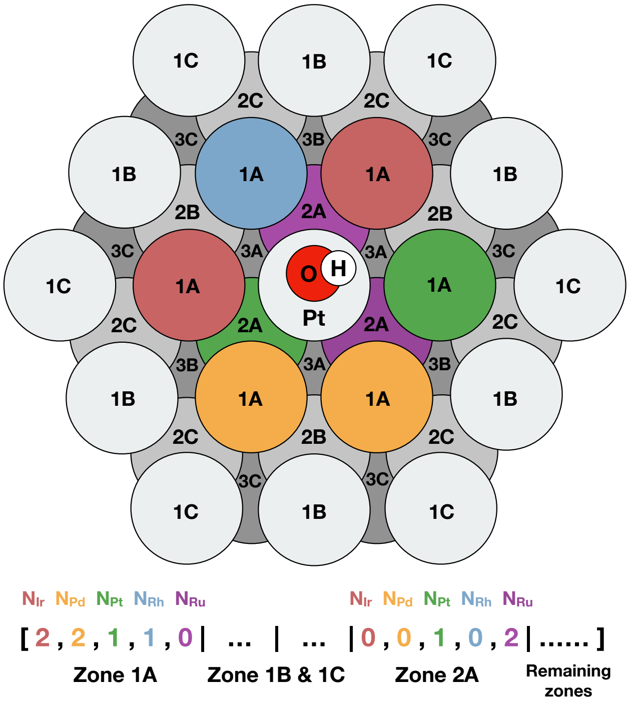

Figure S2: Illustration of the feature vector for adsorbed OH in an on-top Pt-centered site. The nearest atomic positions have been coloured to represent different elements. Each variable in the feature vector corresponds to the number of atoms of a specific element in a specific zone thus accounting for the composition of each zone but not the positions of heterogenous atoms internally in the zones.

A note on linear regression

Because we simulate more samples than the amount of parameters we fit in the multilinear regression we are dealing with an overdetermined system of linear equations. This is complicated by the fact that the zoned feature encoding can reduce several samples of different atomic placements to the same zoned composition which still has different adsorption energies. Therefore our system of linear equations are also inconsistent which means that no exact solution can be found as identical sets of variables can map to different values. This is a common problem when dealing with observational data and is the reason why we fit the set of linear parameters to the values with the least squares method[1] thereby minimizing $||\boldsymbol{A}\vec{w} - \vec{b}||$ and obtaining the best possible fit:

$$\vec{w} = (\boldsymbol{A}^T \boldsymbol{A})^{-1} \boldsymbol{A}^T \, \vec{b} \qquad \text{(S1)}$$

with $\vec{w}$ being the linear coefficients including the intercept, $\boldsymbol{A}$ being the feature matrix and $\vec{b}$ being the target vector of the adsorbtion energies.

Due to the nature of our feature vectors the matrix $(\boldsymbol{A}^T\boldsymbol{A})$ are prone to linear dependency and is therefore singular and non-invertible. Therefore we decompose the feature matrix into a $m\ x\ m$ matrix, $\boldsymbol{Q}$, and an $m\ x\ n$ upper triangle matrix, $\boldsymbol{R}$, with m and n being the number of rows and columns of A. Afterwards we can solve the least squares problem as:

$$\vec{w} = (\boldsymbol{R})^{-1}\boldsymbol{Q}^T\ \vec{b} \tag{S2}$$

This QR decomposition can be done in several ways, the best known being Gram-Schmidt orthogonalization taught in basic linear algebra courses. All linear regression in this work are done with the SKLearn[2] LinearRegression() class, a wrapped version of scipy.linalg.lstsq()[3] which in turn utilizes the Fortran-written standard linear algebra tool of LAPACK[4] to solve the least squares problem. Even though LAPACK QR decomposes the feature matrix using Householder reflections[5] which is a numerically stable method the decomposition is affected by the order of rows in the feature matrix. Hence the linear fits have been performed by shuffling the rows of $\boldsymbol{A}$ before the QR decomposition and least squares fit. This was done iteratively 100 times and the mean linear components were extracted.

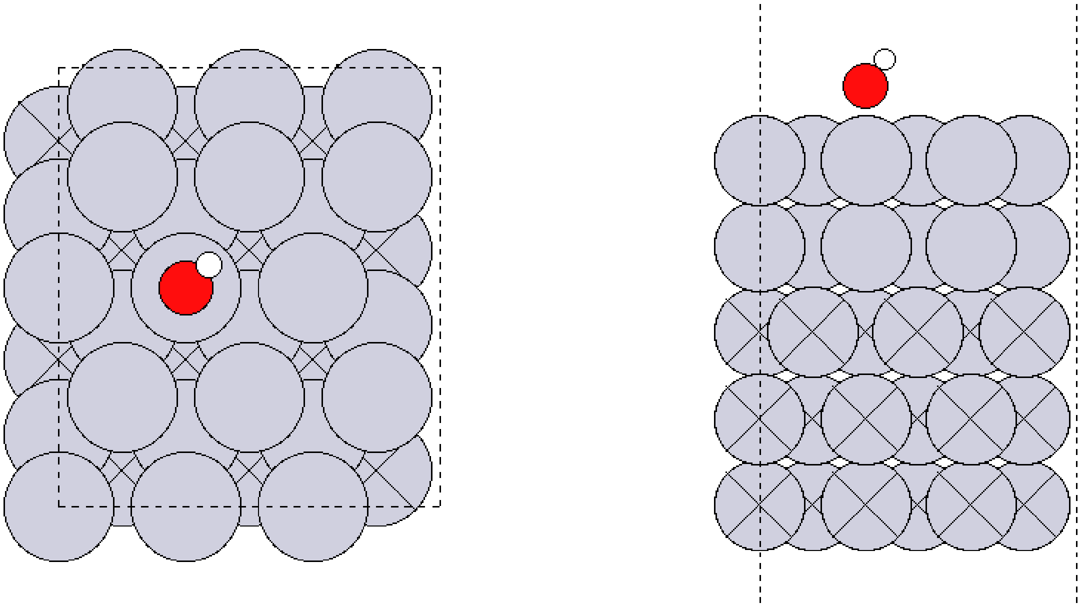

Figure S3: Illustration of adsorbed OH(red and white respectively) in an on-top site on 3x4x5 slab of pure Pt(111)(grey). Due to the slab size restrictions imposed by a 3x4 cell only two-thirds of the atoms in zone 1C and 4C were exchanged for guest elements whereas all the atoms in zone 3A and 3B where exchanged.

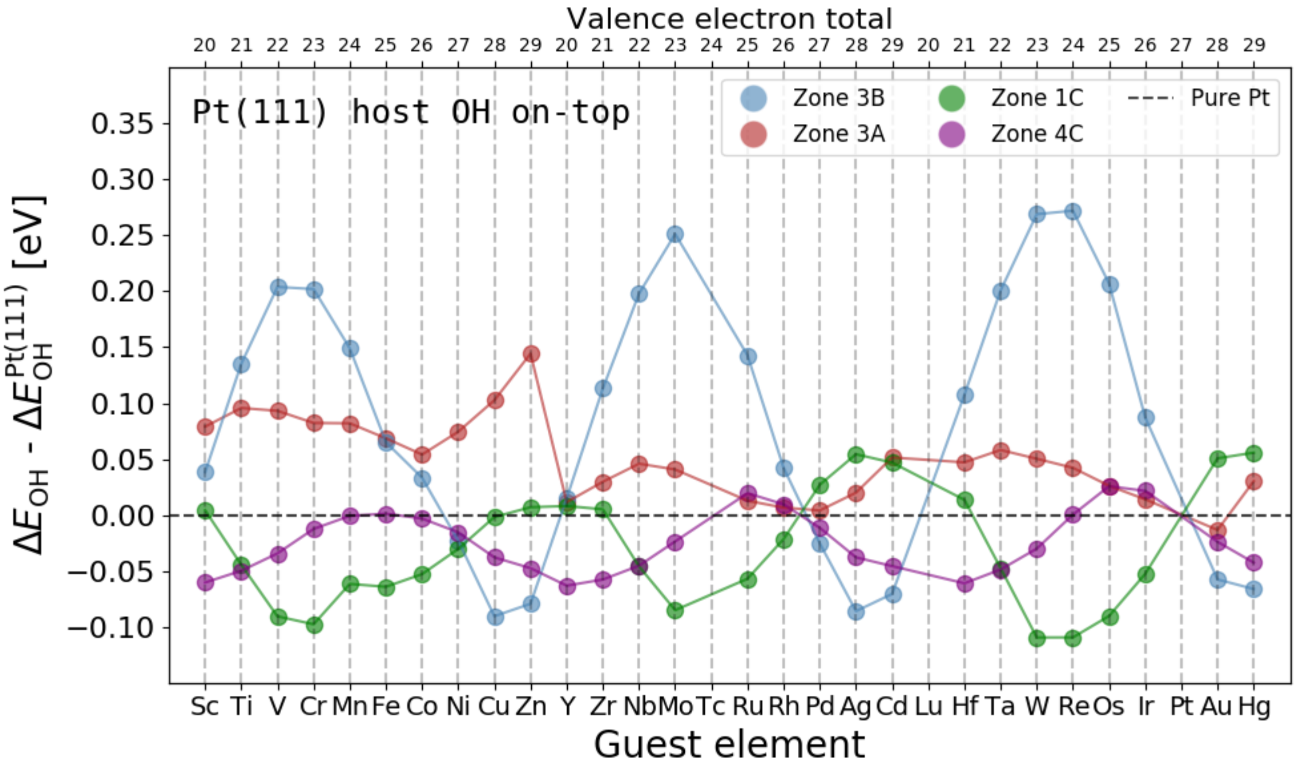

Figure S4: OH adsorption energies on Pt(111) with guest elements inserted in different zones. The atoms of the top two layers of the surface were allowed to relax while the three bottom layers remained at fixed atomic geometry. All adsorption energies are relative to a pure Pt(111) host and after relaxation of atomic geometries. The valence electron total is calculated as the sum of valence electrons of one host atom, one guest atom and the adsorbate atoms. Due to slab size restrictions only two-thirds of the atoms in zone 1C and 4C were exchanged for guest elements whereas all the atoms in zone 3A and 3B where exchanged. The anti-correlation of zone 3B and zone 1C is likely incidental as the minima of adsorption energies are coincides with the smallest lattice parameters of each period. Tensile surface strain in bimetallic alloys has previously been seen to cause shifts towards stronger binding[6]. As the unit cell size were not adjusted between calculations involving different guest elements, the anti-correlation are likely caused by the varying lattice constants of the guest element in zone 1C.

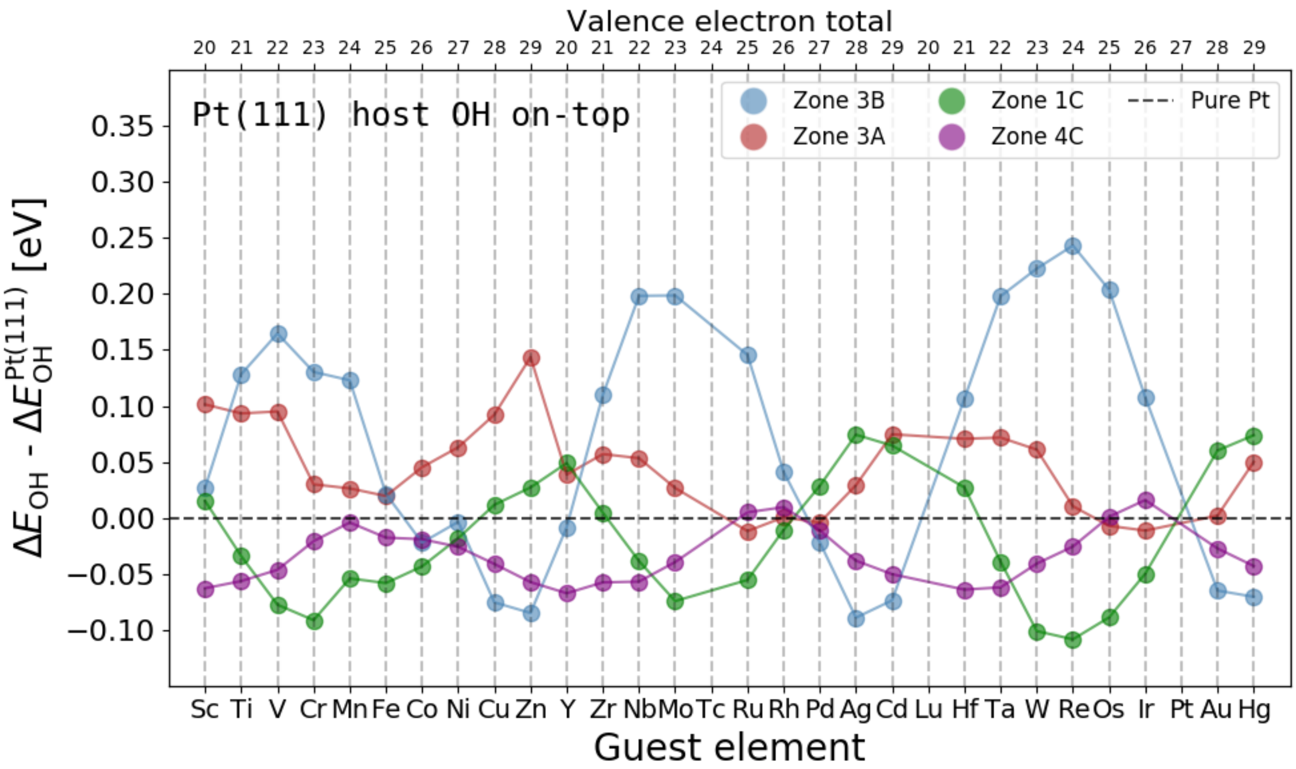

Figure S5: OH adsorption energies on Pt(111) with guest elements inserted in different zones. Atoms in all layers were allowed to relax. All adsorption energies are relative to a pure Pt(111) host. The valence electron total is calculated as the sum of valence electrons of one host atom, one guest atom and the adsorbate atoms. All energies are after relaxation of atomic geometries. Due to slab size restrictions only two-thirds of the atoms in zone 1C and 4C were exchanged for guest elements whereas all the atoms in zone 3A and 3B where exchanged. It is observed that allowing the three bottom layers of the slab to relax do not significantly change the adsorption energy trends.

Relative adsorption energy of Pt(111) host with W guest element		
	RPBE functional	BEEF-vdW functional
Zone 3A	-0.014 eV	-0.017(34) eV
Zone 3B	0.258 eV	0.251(31) eV
Zone 1C	-0.032 eV	-0.027(63) eV
Zone 4C	-0.010 eV	-0.012(34) eV

Table S1: Comparison of adsorption energies for Pt(111) host with on-top OH and W as guest atom in zones 3A, 3B, 1C and 4C. All energies are relative to a pure Pt host.

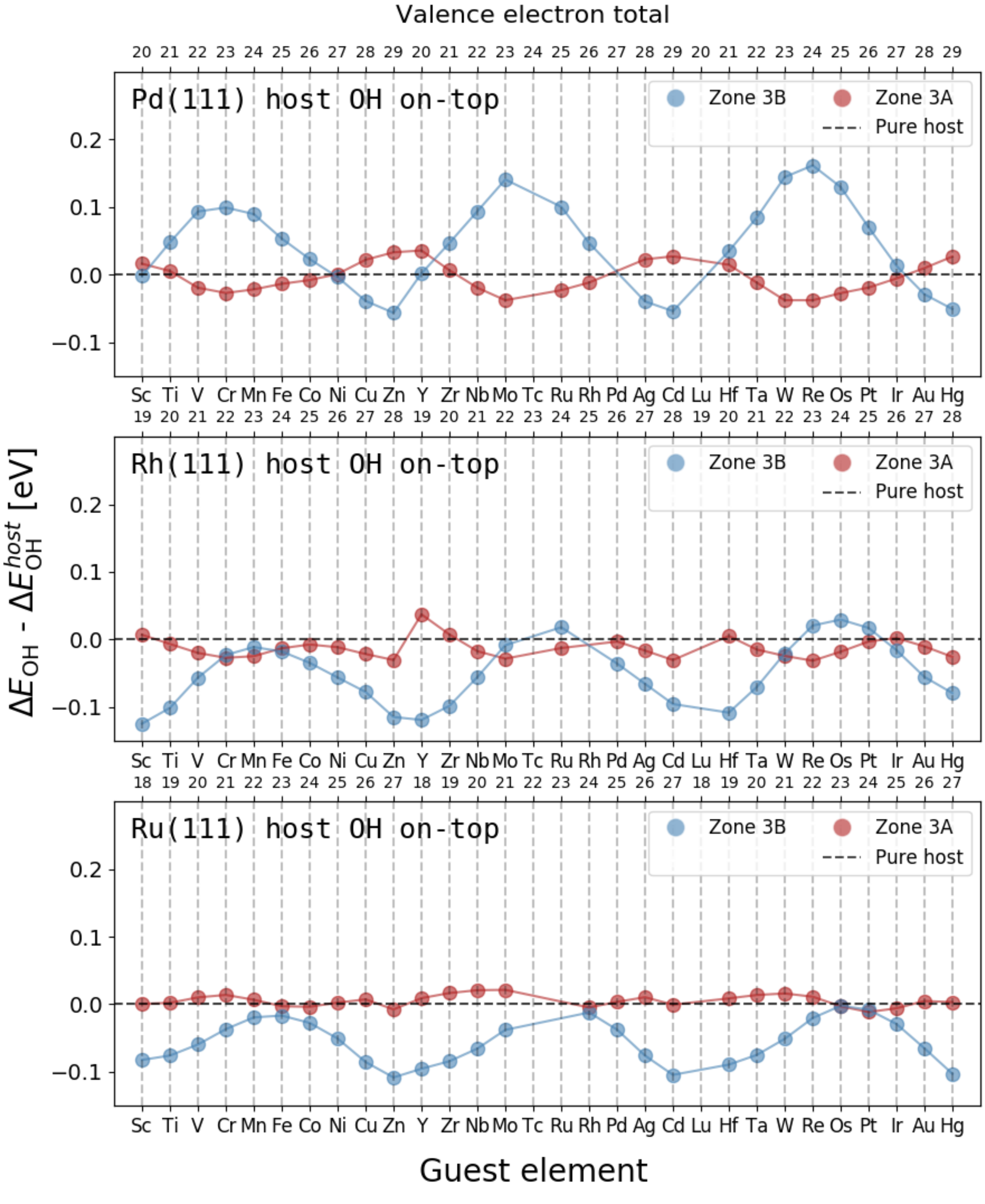

Figure S6: OH adsorption energies on Pd, Rh and Ru with guest elements inserted in different zones. All adsorption energies are relative to a pure host. The valence electron total is calculated as the sum of valence electrons of one host atom, one guest atom and the adsorbate atoms. All calculations were performed at the same fixed atomic geometry.

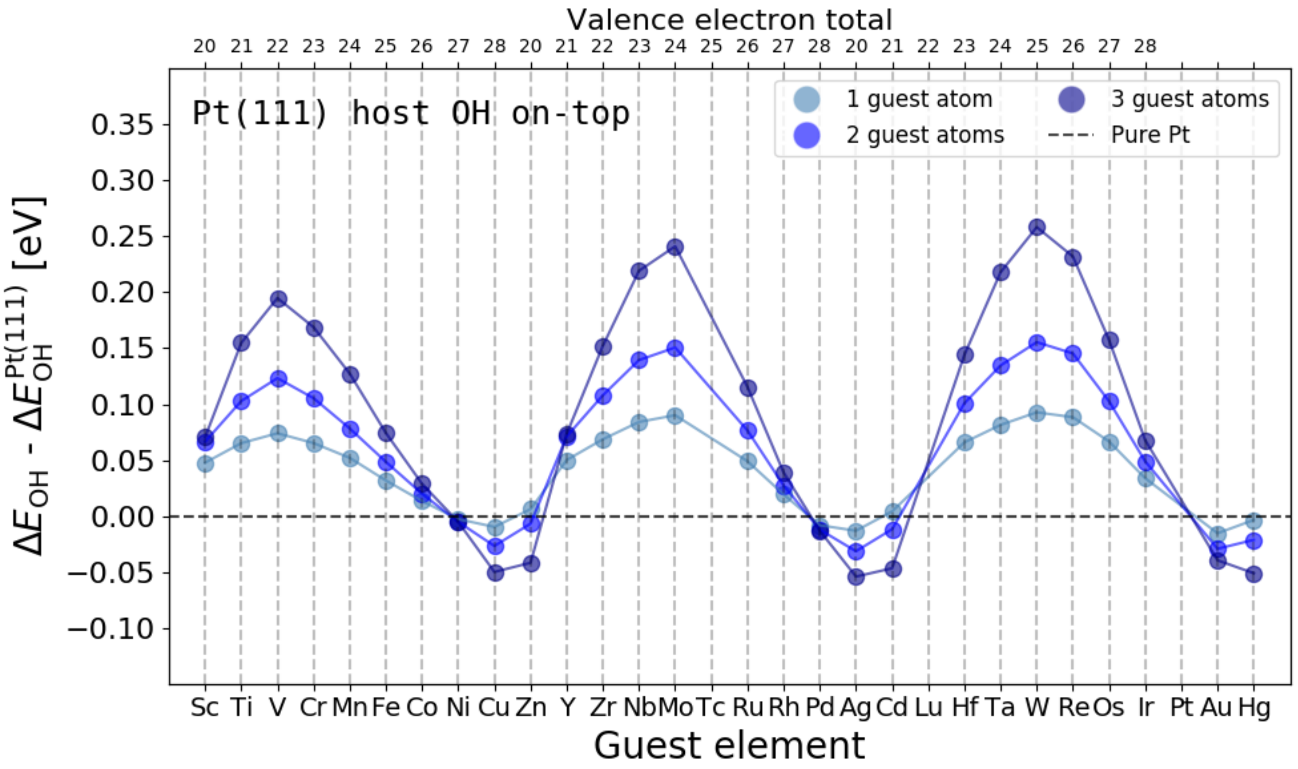

Figure S7: OH adsorption energies on Pt(111) with one, two and three guest atoms inserted in zone 3B. All adsorption energies are relative to a pure Pt(111) host. The valence electron total is calculated as the sum of valence electrons of one host atom, one guest atom and the adsorbate atoms.

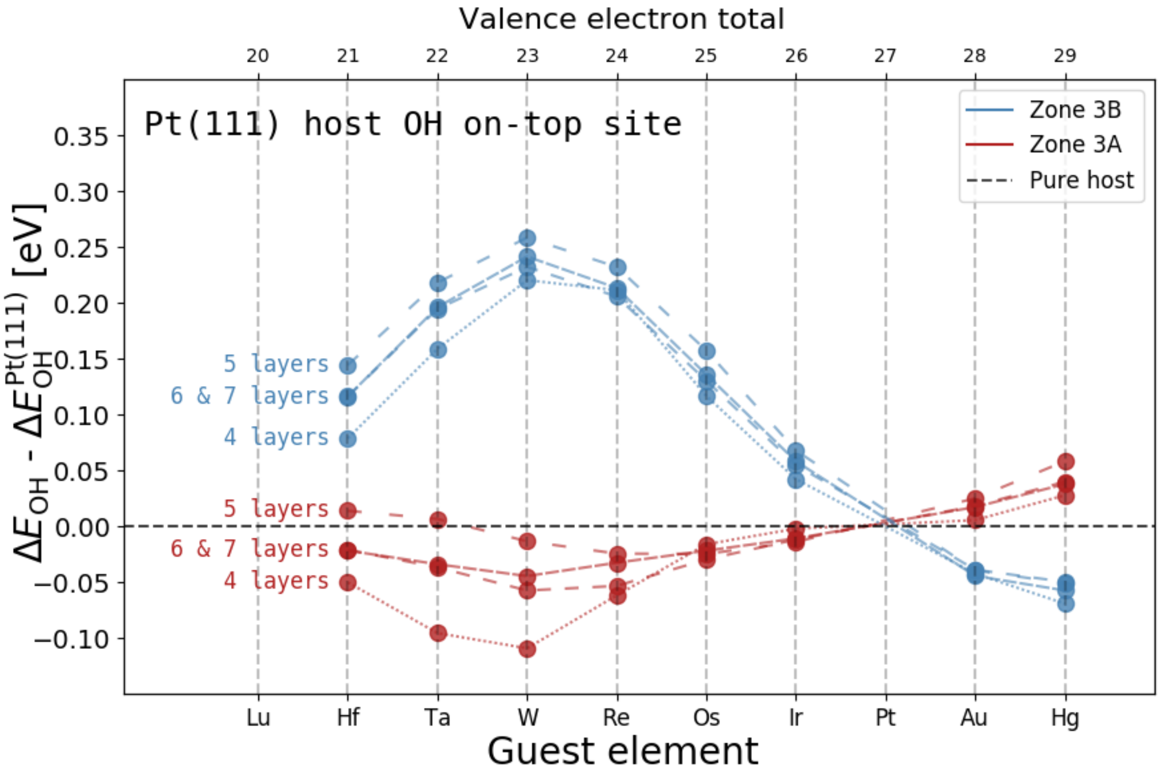

Figure S8: OH adsorption energies on Pt(111) slabs of different thickness with inserted 5d element guests in zone 3A and 3B. Number of layers in each slab is indicated. The valence electron total is calculated as the sum of valence electrons of one host atom, one guest atom and the adsorbate atoms.

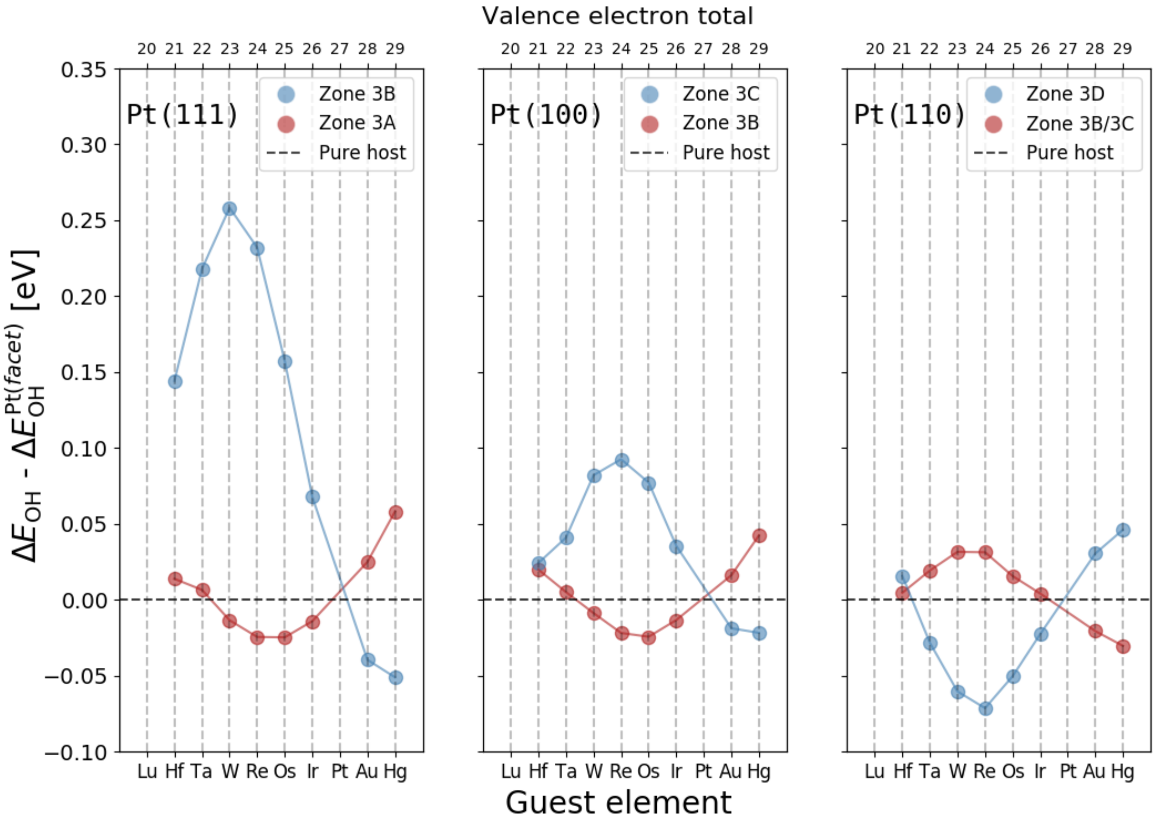

Figure S9: OH adsorption energies on different facets of Pt with inserted 5d guest elements in zones of equivalent direction and distance. Pt(100) and Pt(110) zone layout shown in figure S10. All adsorption energies are relative to a pure Pt host of the same facet. The valence electron total is calculated as the sum of valence electrons of one host atom, one guest atom and the adsorbate atoms. All calculations were performed at the same fixed atomic geometry. It is observed that the influence of zone 3B first diminishes and the switches sign with zone 3A when progressing from fcc(111) over fcc(100) to fcc(110). We speculate that this pattern could emerge because the surface progresses from the closely packed fcc(111) surface to the open fcc(110). Thus, the general reactivity trend of these surfaces progresses from low to high which could interact with the influence of the third layer zones. Further investigation of how this effect manifests in different facets are needed.

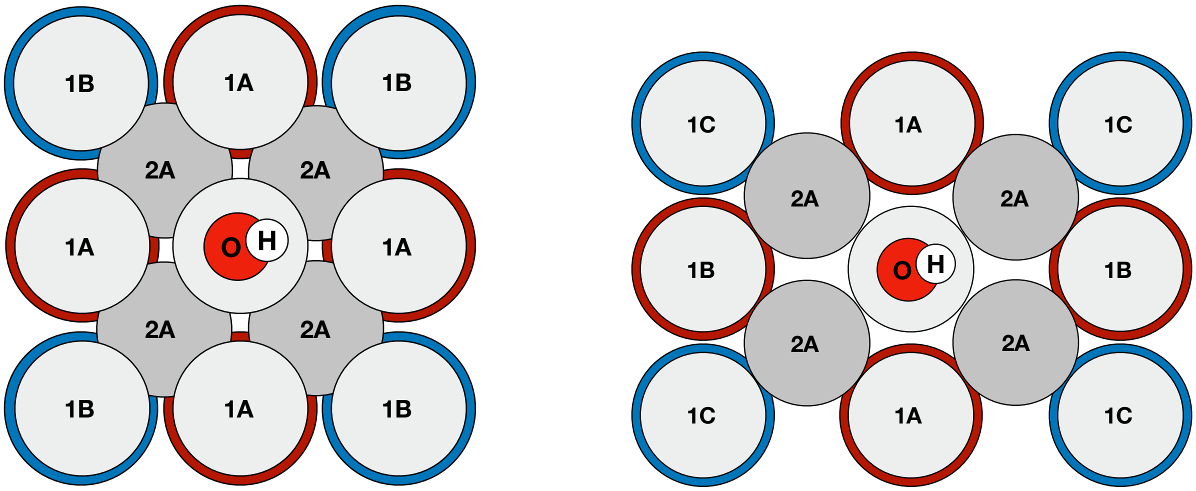

(a) OH on-top on Pt(100)

(b) OH on-top on Pt(110)

Figure S10: Schematic of the OH on-top adsorption site on **a)** Pt(100) and **b)** Pt(110). For Pt(100) the third layer zones of 3B and 3C have been circled with red and blue respectively as these reside directly beneath zone 1A and 1B. For fcc(110) both zone 3B and 3C are marked with red and zone 3D are marked in blue. For both facets the zone marked with blue have a similar favorable vector of electronic interaction as zone 3B in fcc(111).

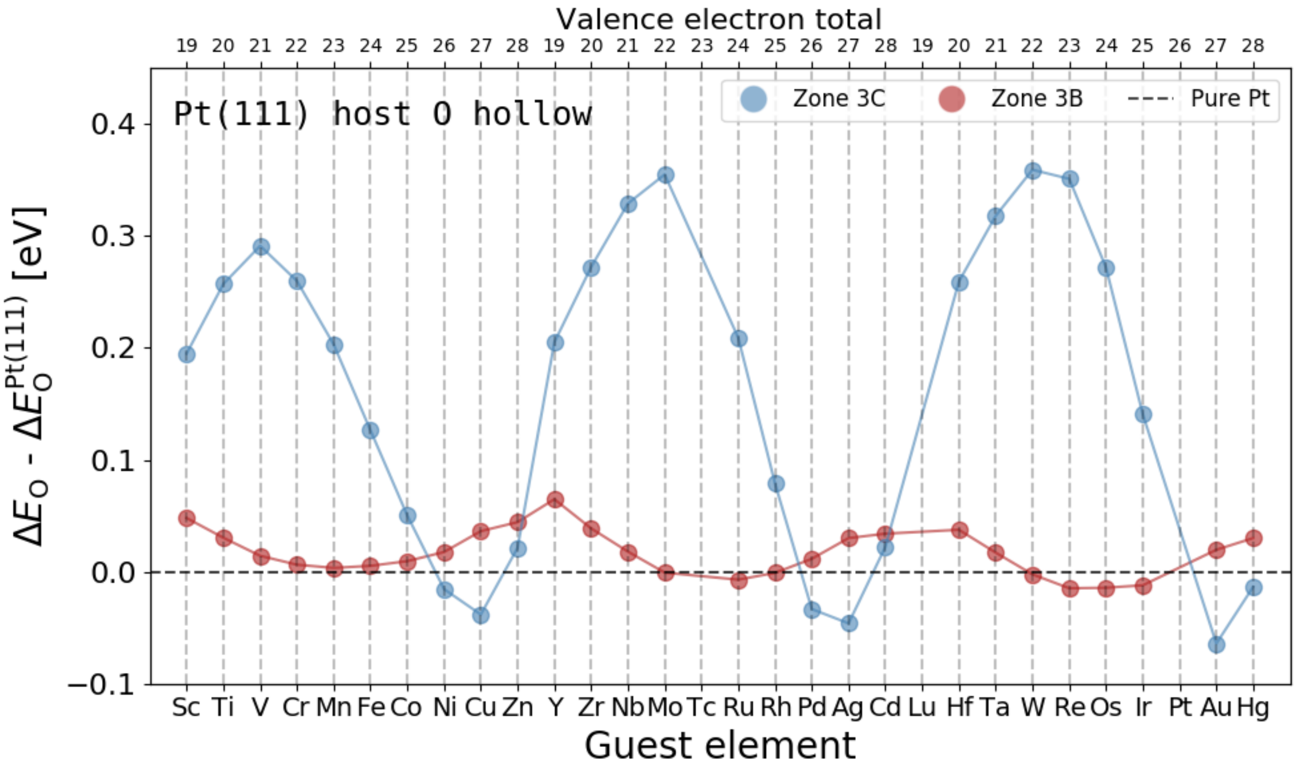

Figure S11: O adsorption energies on Pt with guest elements in zones 3B and 3C. All adsorption energies are relative to a pure Pt(111) host. The valence electron total is calculated as the sum of valence electrons of one host atom, one guest atom and the adsorbate atoms. All calculations were performed at the same fixed atomic geometry and three atomic positions in zone 3C and 3B was occupied with guest elements. The increased change in adsorption energy compared to the host/guest calculations of OH are due to the higher order of the O to surface bond[7].

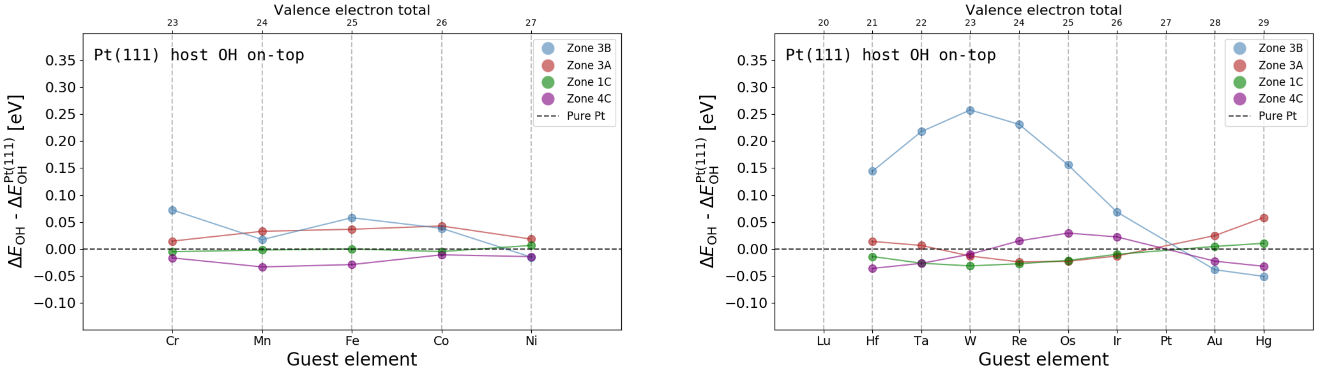

(a) The Cr-Mn-Fe-Co-Ni family of 3d metals

(b) 5d metals

Figure S12: Spin-polarized OH adsorption energies on Pt with guest elements inserted in different zones. All adsorption energies are relative to a pure Pt(111) host. The valence electron total is calculated as the sum of valence electrons of one host atom, one guest atom and the adsorbate atoms. **a)** The Cr-Mn-Fe-Co-Ni family of 3d elements. It is observed that the spin-polarization quenches most of the electronic interaction, especially for Cr and Mn, which will require further investigation. **b)** For the non-magnetic 5d elements the magnitude of the effect is consistent with the non-spin-polarized calculations

.

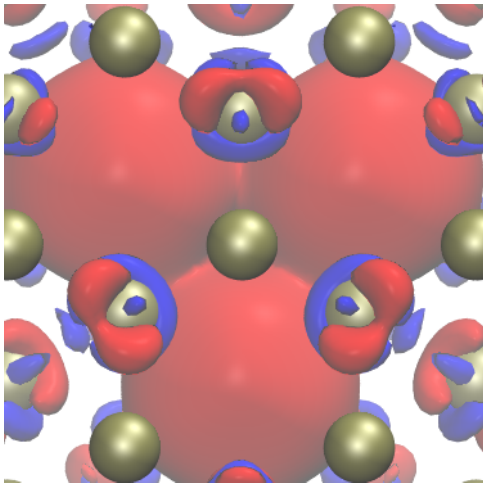

(a) Top view of Ru in zone 3A.

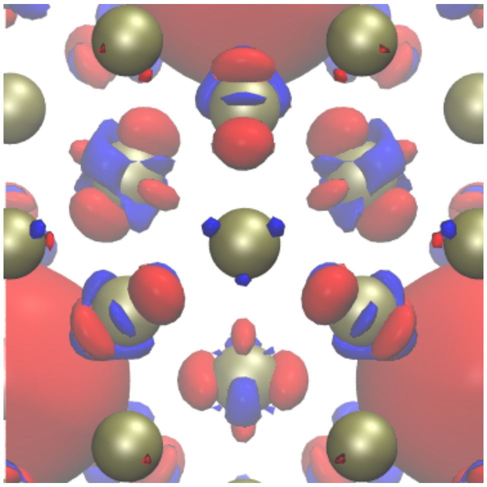

(b) Top view of Ru in zone 3B.

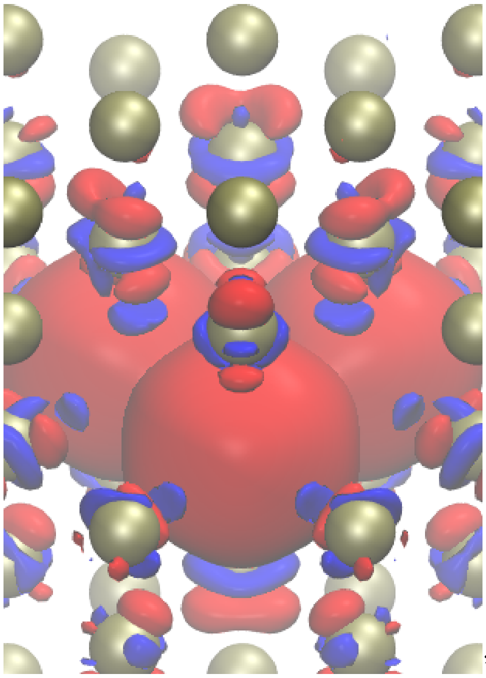

(c) Side view of Ru in zone 3A.

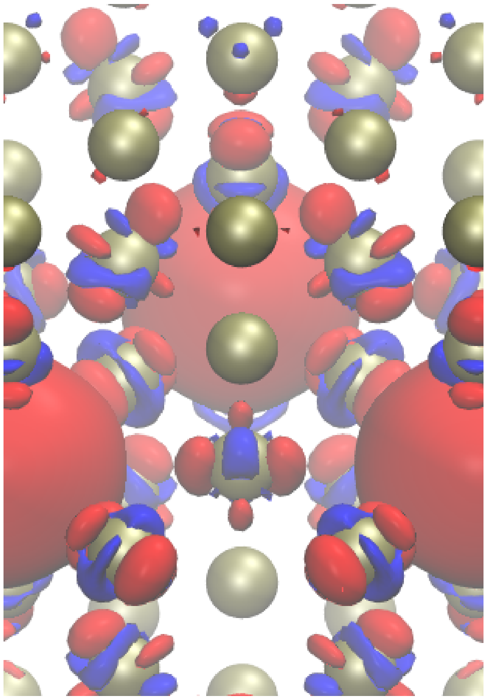

(d) Side view of Ru in zone 3B.

Figure S13: Top and side views of isosurfaces of charge density differences between a pure Pt(111) host and Ru guest atoms in zone 3A (**a)** and **c)**) and zone 3B (**b)** and **d)**). Red corresponds to subtracted charge density and blue to added density. Isovalue set to $\pm\, 2 \cdot 10^{-3}$ e/Å^3.

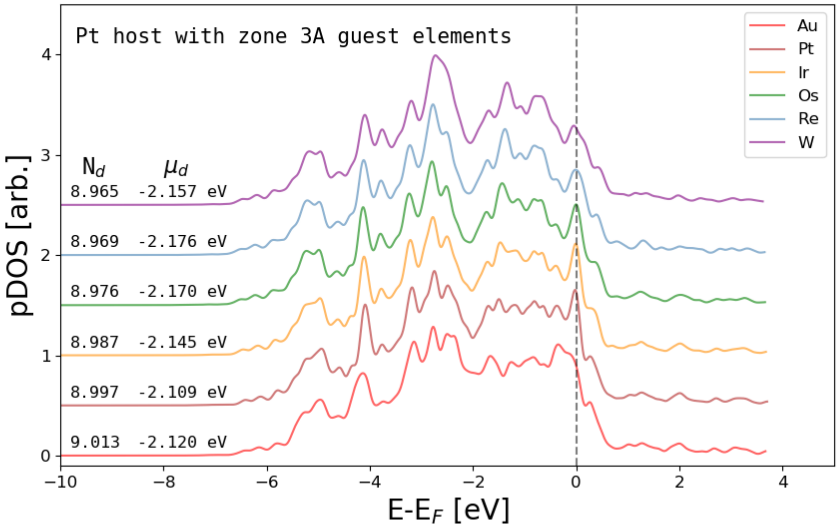

(a) Projected density of states.

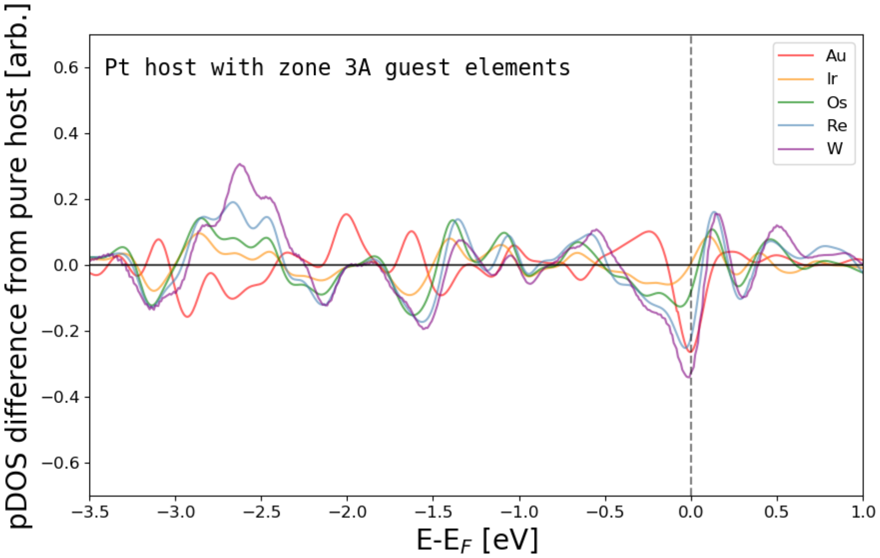

(b) Residual plot of pDOS difference.

Figure S14: a) Projected density of d states for the binding atom in the Pt(111) host with different guest elements in zone 3A with the Fermi level at 0 eV and off-sets of 0.5 eV. Number of d-electrons on the binding atom, N_d, and the d-band center, μ_d, are displayed. **b)** Zoomed residual plot of the difference between a pure host slab and inserting guest elements.

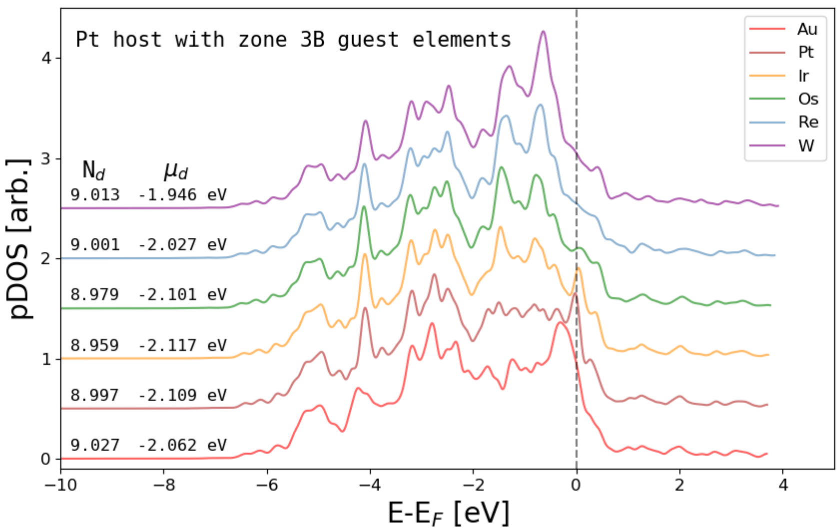

(a) Projected density of states.

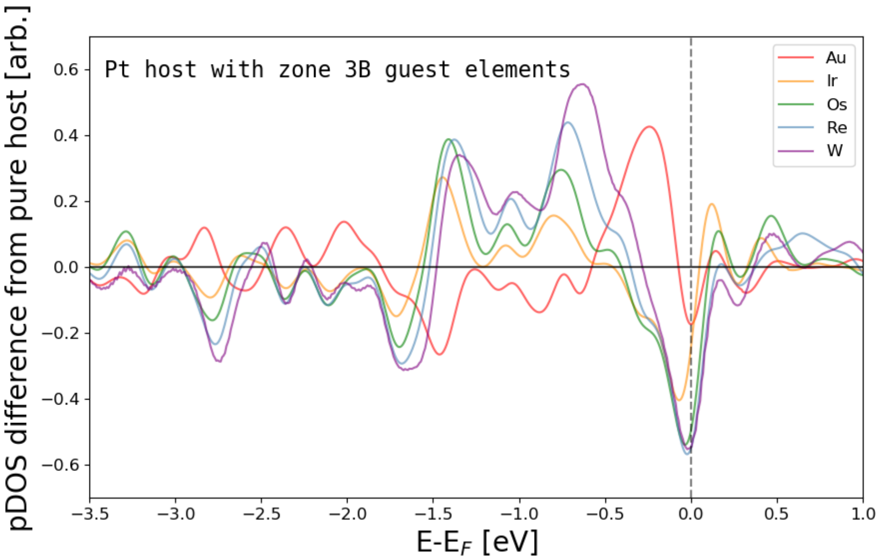

(b) Residual plot of pDOS difference.

Figure S15: a) Projected density of d states for the binding atom in the Pt host with different guest elements in zone 3B with the Fermi level at 0 eV and off-sets of 0.5 eV. Integrated number of d-electrons on the binding atom(N_d) and the d-band center(μ_d) are displayed. As the bond to the adsorbate gets sequentially weaker in this series we see that the d-band center does not correlate with the change in ΔE. **b)** Zoomed residual plot of the difference between a pure host slab and inserting guest elements. It is clear that there are areas of sequential and systematic change to the d-band shape which could reflect the changes in bond strength. Particularly, the rising peaks around -0.5 eV and -1.5 eV could result in increased overlap with the antibonding orbitals of the adsorbate, Conversely, the diminishing peaks at -1.6 eV and -2.75 eV could lessen the overlap with the bonding orbitals.

References

[1] M. Planitz, *Math. Gaz.* **1979**, *63* 181.

[2] F. Pedregosa, G. Varoquaux, A. Gramfort, V. Michel, B. Thirion, O. Grisel, M. Blondel, P. Prettenhofer, R. Weiss, V. Dubourg, J. Vanderplas, A. Passos, D. Cournapeau, M. Brucher, M. Perrot, E. Duchesnay, *J. Mach. Learn. Res.* **2011**, *12* 2825.

[3] E. Jones, T. Oliphant, P. Peterson, et al., Scipy: Open source scientific tools for python, URL `http://www.scipy.org/`, Accessed: 2019-10-21.

[4] E. Anderson, Z. Bai, C. Bischof, S. Blackford, J. Demmel, J. Dongarra, J. Du Croz, A. Greenbaum, S. Hammarling, A. McKenney, D. Sorensen, *LAPACK User Guide*, Society for Industrial and Applied Mathematics, **1999**.

[5] Qr factorization in lapack, URL `https://www.netlib.org/lapack/lug/node69.html`, Accessed: 2019-10-21.

[6] K. J. Andersson, F. Calle-Vallejo, J. Rossmeisl, I. Chorkendorff, *J. Am. Chem. Soc.* **2009**, *131*, 6 2404.

[7] F. Abild-Pedersen, *Catal. Today* **2016**, *272* 6.
